# Age is not just a number: How incorrect ageing impacts close‐kin mark‐recapture estimates of population size

**DOI:** 10.1002/ece3.11352

**Published:** 2024-06-04

**Authors:** Felix T. Petersma, Len Thomas, Danielle Harris, Darcy Bradley, Yannis P. Papastamatiou

**Affiliations:** ^1^ Centre for Research into Environmental and Ecological Modelling University of St Andrews St Andrews UK; ^2^ Bren School of Environmental Science & Management University of California Santa Barbara California USA; ^3^ Department of Biological Sciences, Institute of Environment Florida International University North Miami Florida USA

**Keywords:** abundance estimation, *Carcharhinus amblyrhynchos*, Grey reef shark, measurement error, simulation, von Bertalanffy growth function

## Abstract

Population size is a key parameter for the conservation of animal species. Close‐kin mark‐recapture (CKMR) relies on the observed frequency and type of kinship among individuals sampled from the population to estimate population size. Knowledge of the age of the individuals, or a surrogate thereof, is essential for inference with acceptable precision. One common approach, particularly in fish studies, is to measure animal length and infer age using an assumed age‐length relationship (a ‘growth curve’). We used simulation to test the effect of misspecifying the length measurement error and the growth curve on population size estimation. Simulated populations represented two fictional shark species, one with a relatively simple life history and the other with a more complex life history based on the grey reef shark (*Carcharhinus amblyrhynchos*). We estimated sex‐specific adult abundance, which we assumed to be constant in time. We observed small median biases in these estimates ranging from 1.35% to 2.79% when specifying the correct measurement error standard deviation and growth curve. CI coverage was adequate whenever the growth curve was correctly specified. Introducing error via misspecified growth curves resulted in changes in the magnitude of the estimated adult population, where underestimating age negatively biased the abundance estimates. Over‐ and underestimating the standard deviation of length measurement error did not introduce a bias and had negligible effect on the variance in the estimates. Our findings show that assuming an incorrect standard deviation of length measurement error has little effect on estimation, but having an accurate growth curve is crucial for CKMR whenever ageing is based on length measurements. If ageing could be biased, researchers should be cautious when interpreting CKMR results and consider the potential biases arising from inaccurate age inference.

## Graphical abstract

Close‐kin mark‐recapture is a novel method to estimate animal abundance based on the type and frequency of observed kinship. Through simulation, we tested the effects of misspecified age and length measurement on close‐kin mark‐recapture population size estimates. We found that length measurement had negligible effect, but that the method was sensitive to biased ageing.

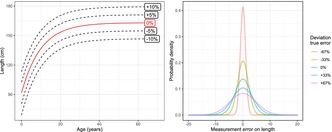

## 1 INTRODUCTION

Close‐kin mark‐recapture (CKMR) is a method for estimating population size and other key parameters such as fecundity (and population growth and survival rates) using data on the relatedness of individuals sampled from the population (Bravington, Skaug, & Anderson, 2016; Skaug, 2001). The key rationale is that small populations will tend to contain a higher proportion of closely related individuals than large populations.

One of the main advantages of CKMR over capture‐recapture (Otis et al., 1978) and its extensions such as spatial capture‐recapture (Borchers & Efford, 2008) is that it can be applied in cases when sampling is necessarily lethal, such as fisheries, and when physical recaptures are rare or impossible, where alternative metrics are often relative (e.g., catch‐per‐unit‐effort) and potentially unreliable (Bravington, Grewe, & Davies, 2016; Casas & Saborido‐Rey, 2023). This is because CKMR does not require the recapturing of individuals, but rather their genetic markers. Offspring share genetic information with their parents (hence ‘kin’), thus they ‘mark’ their parents when born; through modern genetics we can compare sampled individuals with one another to see if these marks are ‘recaptured’. So far, CKMR has been developed for parent‐offspring pairs (POPs; e.g., Bravington, Grewe, & Davies, 2016; Ruzzante et al., 2019; Trenkel et al., 2022), half‐sibling pairs (HSPs; e.g., Hillary et al., 2018; Bravington et al., 2019; Patterson et al., 2022), and the combination of both (e.g., Bradford et al., 2018). The rise in popularity of the method has become clear from an increase in published studies involving CKMR, although the total number of applications is still small (Delaval et al., 2023). Most of the applications up to this point involved marine or aquatic species. Several salmonids have been studied (Prystupa et al., 2021; Ruzzante et al., 2019; Wacker et al., 2021), as well as large pelagic species such as southern bluefin tuna (*Thunnus maccoyii*; Bravington, Grewe, & Davies, 2016) and the pelagic bluefin tuna (*Thunnus orientalis*; Tsukahara et al., 2023), and a variety of elasmobranchs such as white sharks (*Carcharodon carcharias*; Hillary et al., 2018), lemon sharks (*Negaprion brevirostris*; Swenson et al., 2024), thornback rays (*Raja clavata*; Trenkel et al., 2022), blue skates (*Dipturus batis*; Delaval et al., 2023), and grey nurse sharks (*Carcharias taurus*; Bradford et al., 2018). The Christmas Island ying‐fox (*Pteropus natalis*; Lloyd‐Jones et al., 2023) and the yellow fever mosquito (*Aedes aegypti*; Sharma et al., 2022) were the only terrestrial species that we could identify in published CKMR studies to date.

CKMR with POPs can estimate the size of the entire adult population, whereas with HSPs only the *breeding* adult population is estimated, for example, post‐reproductive adults are ‘invisible’ for the method (Bradford et al., 2018). Here, we focus only on POPs. For any comparison between two individuals, the probability that a potential offspring truly is the offspring of the parent is inversely related to the number of mature animals alive in the birth year of the offspring. Probabilities of finding a kin pair are expressed as a function of the expected relative reproductive output (ERRO) of the parent in the year that the offspring was conceived. This approach is parent‐centric, as it starts from the point that the parent is sampled and then formulates a probability for a PO relationship (an alternative, offspring‐centric formulation was proposed by Skaug (2017)).

In the simplest scenario, the probability of any adult being the parent of a juvenile reduces to two over the number of potential parents, assuming a 50:50 sex ratio; in reality, this probability is often more complicated, for example, when reproductive output is related to age, or when there is stock structure or population trend. To use relatedness to estimate adult population abundance with acceptable precision, it is therefore essential to accurately age the studied animals because birth year is derived from their age. Accurate ageing can be challenging: for example, epigenetic ageing requires calibration using individuals of known age (De Paoli‐Iseppi et al., 2017; Polanowski et al., 2014), which is not always possible; ageing via otoliths, which are calcium carbonate structures in the inner ear, can be relatively accurate (Campana, 2001) but requires lethal sampling and is only possible for animals that have otoliths (and sharks are not among those); and ageing by counting the dental or cementum growth layer groups in teeth is not necessarily lethal and commonly used for (marine) mammals (Hohn, 2009, Chapter 9), but cannot be applied to fish species. Sharks can be aged from their vertebrae, but this is a lethal procedure and can be biased in various ways or even unusable depending on the species (Burke et al., 2020). Alternatively, length can be used to infer age through growth curves, which seems appealing as length is often recorded during sampling. Accurate estimates for growth curves of the studied species are not always available, however, and age as a function of length (age‐at‐length) can vary substantially between populations of the same species (e.g., Bradley, Conklin, Papastamatiou, McCauley, Pollock, Kendall, et al., 2017). Moreover, length measurements often involve measurement error. Swenson et al. (2024) studied the effects of ageing error from incorrect length measurement through simulation and found that incorrect ageing can induce substantial bias in CKMR parameter estimates. Various degrees of error were added to the true lengths of individuals, after which these were converted to ages using a von Bertalanffy growth curve. These ‘incorrect’ ages were then used as inputs for the CKMR model without explicitly modelling the length measurement error.

Simulation is an important tool to assess the robustness of statistical methods to violations of model assumptions (DiRenzo et al., 2023) and their performance more generally (Morris et al., 2019). Through simulation, Conn et al. (2020) studied the effects of unmodelled spatial heterogeneity on CKMR estimation and found that this can induce a negative bias in the abundance estimates; Sévêque et al. (2024) found that fitting overly simplistic CKMR models (that do not account for complex life‐history traits or selective sampling) can cause biases in survival and estimates in non‐trivial directions; and Waples and Feutry (2022) showed, among other things, that age‐specific vital rates can bias abundance estimates from CKMR. We follow an agent‐based simulation approach similar to Swenson et al. (2024) to explore the effects of incorrect ageing on the CKMR adult abundance estimator. Unlike Swenson et al. (2024), our model does not assume length (and thus age) to be perfectly known but rather we explicitly account for the measurement error on lengths.

It is often important for demographic modelling to account for the uncertainty in the age estimates, especially when sampling probabilities depend on the age of individuals (i.e., when there is ‘selectivity’), which is fundamental to fishing (Vasilakopoulos et al., 2020). Correctly accounting for ageing error is therefore still an active part of fisheries research (e.g., Hulson & Williams, 2024). Fournier and Archibald (1982) showed how ageing error in catch‐at‐age data can be accounted for as long as the ageing error is known. Later, Richards et al. (1992) developed statistical methodology to account for ageing error when the error is unknown, using multiple readings of fish. We are unaware of any CKMR studies in which ageing error is directly modelled and estimated. Bravington et al. (2019) accounted for the uncertainty in ageing by first fitting a known‐age CKMR model to the data and then refitting the model ten times, resampling ages from the age‐at‐length curve each time. In our simulation, ageing error is introduced in two ways: (i) through misspecified growth curves, and (ii) through incorrect length measurements, that is, measurement error. In reality, error could also be (and almost surely is) introduced within a population through natural variation in length‐at‐age, for example, as a function of genetic and environmental factors. We assume that all individuals follow the growth curve perfectly; however, one could readily interpret the length measurement error as the joint error of length measurement and length‐at‐age variation, or even solely as length‐at‐age variation if that is more appropriate for a particular case study. We assume ageing error from incorrect length measurements to be known and explicitly account for it in our model (Bravington, Skaug, & Anderson, 2016, Section 3.1.4).

The research presented in this manuscript is centred around two fictional shark species that are based on a grey reef shark population (*Carcharhinus amblyrhynchos*) at Palmyra Atoll, in the central Pacific Ocean (Bradley, Conklin, Papastamatiou, McCauley, Pollock, Pollock, et al., 2017; Papastamatiou et al., 2018). This motivating case study consists of genetic samples that were collected from this population in 2013 and 2014. One fictional species is a simplification of the real species (hereafter referred to as the ‘simple species’) and was included to test the basic performance of the model. The other fictional species has more realistic life history traits (hereafter referred to as the ‘complex species’) and was included to more closely match a real empirical study. We also compare the results for both species. It is paramount to first explore the feasibility of CKMR, for example through simulation, before committing the resources and time required for the correct collection and genetic analysis of the samples. Moreover, the findings will be relevant to other CKMR studies when age is uncertain.

## 2 MATERIALS AND METHODS

We first present our setup of the simulations for the two fictional shark species. Simulated time series are 100 years long, with sampling occurring in the final 2 years (mimicking the 2 years of sampling in the Palmyra Atoll case study). Following that, we present the POP‐based CKMR models for our two species using these 2 years of data, followed by our estimation method and performance diagnostics. We assume that kinship relationships are known with certainty; in real‐life situations, one often needs to account for uncertainty in this process (Bravington, Skaug, & Anderson, 2016). All variables and quantities used in this study are summarised in Table 1. Code for the simulation and fitting of models was written in R 4.3.2 and C++14, where the latter was linked to R through Rcpp 1.0.12 (Eddelbuettel, 2013; R Core Team, 2023).

**TABLE 1:** Summary of notation.

Symbol	Description	Type
*General*		
n	Number of sampled individuals (individuals can be sampled more than once)	Observed
p	Detection probability of an individual	Function
f	Probability density/mass function	Function
K	Kinship category	Latent/observed
N	Adult abundance	Parameter
*Quantities related to a captured individual*		
y	Birth year	Latent
c	Capture/sampling year	Observed
ℓ	Length (when captured)	Observed
$\sigma_{\ell }$	Standard deviation of the length measurement error	Parameter
a	Age (when captured)	Latent
s	Sex	Observed
z	Vector of observed covariates at time of capture/sampling	Observed
*Population dynamics and demography*		
ϕ	Survival probability from 1 year to the next	Parameter
r	Growth rate parameter from 1 year to the next	Parameter
α	Age of maturity	Parameter
*Subscript*		
i,j	Individual i and j	
♀, ♂	Sex, either female or male	
t	Year	
m	Simulation index	

### 2.1 Simulation

**FIGURE 1:**
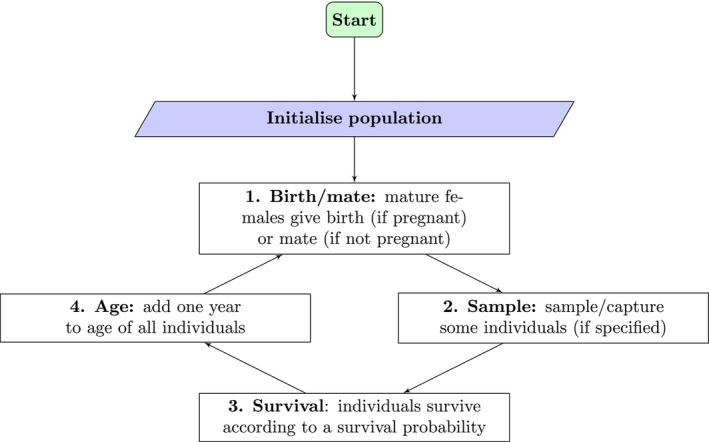
Flowchart representing the different stages of the life‐cycle for the simulation. A population is initialised at the start of a simulation. Following that, it loops through stage 1–4 every year the simulation runs.

We used stochastic individual‐based (‘agent‐based’) simulation. Two different ‘species’ were simulated separately, one with simple life history characteristics, and one with a more complex life history. For each simulation, sampling in the last 2 years was random, and mating occurred at random as well, that is, mothers and fathers were matched at random, where all non‐gestating mothers mated and mature males could father multiple litters in the same mating cycle. Females of the simple species always produced two offspring, whereas the litter size for the complex species ranged from 3 to 6, with equal probability. Females of the simple species reproduced every year as gestation was negligible; females of the complex species gestated for a year and therefore reproduced every other year. Newborns had age zero and sex was assigned at random with an expected 50:50 sex ratio. The survival process was Bernoulli where the annual survival probability ϕ was the same for all ages and sexes, but different between the two species and empirically set at a level that resulted in the yearly population growth rate equalling approximately one, that is, no growth. Natural mortality was the only source of mortality we considered, and all individuals that reached the maximum age perished at the next survival event, that is, animals could go through at most $a_{\max}+1$ yearly cycles. The maximum age for sharks of the simple species was 19 years and 63 years for the complex species, where the latter matches the results from Bradley, Conklin, Papastamatiou, McCauley, Pollock, Kendall, et al. (2017). For a given species, all individuals of the same sex matured at the same age: males and females in the simple species matured at 10 years old, whereas in the complex species males matured at 17 years and females matured at 19 years of age. The length of an animal was the same for all individuals of a certain age, irrespective of sex and species. After the initialisation of a population in year zero, the simulation looped through four distinct events: a birthing/mating event, a sampling event (only in the final 2 years of the simulation), a survival event, and an ageing event (Figure 1).

For both species, we ran the simulations for 100 years, to ensure that all animals of the initial populations would have died off. Every simulation started with 8500 individuals to stay close to the population size estimate of 8433 by Bradley, Conklin, Papastamatiou, McCauley, Pollock, Pollock, et al. (2017), with an expected 50:50 sex ratio. At each sampling event, 375 individuals were randomly and non‐lethally sampled, where re‐captures were possible between sampling events. This resulted in at most 750 unique sampled individuals across the 2 years of sampling, which is of a similar scale as the number of genetic samples available in the motivating case study. All 750 samples were retained for analysis as there was no particular reason to exclude recaptures, unlike, for example, Hillary et al. (2018), where duplicate samples were excluded from the analysis to avoid them aliasing as half‐sibling pairs. For every sampled individual, the age, year of capture, and sex were recorded; the true length was derived through a von Bertalanffy growth function (VBGF; von Bertalanffy, 1938; Francis, 1988), that was specified as

(1) $$l\left(a\right)=l_{\infty }\times \left(1-e^{-k\left(a-a_{0}\right)}\right),$$

where $\ell_{\infty }=163$ cm is the asymptotic length, $a_{0}=-8.27$ the theoretical age at length zero, and k=0.0554 denotes the growth coefficient. These values match the estimates of the best model in table 2 of Bradley, Conklin, Papastamatiou, McCauley, Pollock, Kendall, et al. (2017). Gaussian noise was added to reflect (symmetric) length measurement error with variance $\sigma_{\ell }^{2}=2.89^{2}$, with over‐ and underestimates being equally likely, after which this ‘observed’ length was rounded to the nearest integer. Based on these parameters, we generated 1000 different realisations of a 100‐year‐long population history for each species, using functions based on those from the fishSim‐package (Baylis, 2022).

### 2.2 POP‐based estimator

We developed estimators for both populations based only on POPs. Any other possible genetic relationship (such as half‐sibling or self‐capture) was categorised as ‘not a POP’. CKMR models are generally fitted through a likelihood (function), which is constructed from the joint distribution of all pairwise comparisons between the samples, that is, the product of approximately $n\left(n-1\right)/2$ Bernoulli trials for a POP, where n is the number of samples. We only consider pairwise comparisons and treat these as independent, whereas they clearly are not: an offspring can only have one parent of each sex. Because we ignore these higher dependencies, our likelihood is not a *true* likelihood but rather a *pseudo*‐likelihood. Working with such a likelihood should not affect the point estimates but could affect other properties of likelihood‐based estimation, such as variance estimation, although this effect is likely minor or even negligible provided that a small proportion of the total population is sampled, that is, n≪N (Bravington, Skaug, & Anderson, 2016; Skaug, 2001). Because length is measured with error and age is inferred from length, age is uncertain and hence we cannot assume directionality in the comparison, that is, who is the parent and who is the offspring. Therefore, for any comparison for individual i and *j*, we test both directions (parent‐offspring and offspring‐parent), denoted PO/OP. In practice, we tend to optimise the logarithm of the pseudo‐likelihood, the so‐called ‘pseudo‐log‐likelihood’, as this is generally easier to work with and numerically more stable. Our pseudo‐log‐likelihood is given by

(2) $$\begin{array}{l} \log L_{P}\left(\theta \text{|}x\right)=l_{P}\left(\theta \text{|}x\right)=\\ \underset{i}{\sum }\underset{j}{\sum }\log\left\{\mathrm{Pr}{\left(K_{ij}=PO/OP\text{|,}z_{i}\text{|,}z_{j}\right)}^{\omega_{ij}}(1-\mathrm{Pr}(K_{ij}=PO/OP\text{|,}z_{i}\text{|,}z_{j}))1-\omega_{ij}\right\}, \end{array}$$

where θ is the parameter vector, x denotes the observed data, $K_{\mathrm{ij}}$ is the kinship between i and j, Pr is the probability function, $\omega_{\mathrm{ij}}$ is an indicator that is 1 if the kinship between i and j is observed to be PO/OP and 0 otherwise, and z denotes the information recorded about a captured individual, such as length. Age is required to calculate the probability of observing kinship, and therefore we sum over all potential ages for i, j and multiply by the probability density of that age given the measured length, $f\left(a|l^{*}\right)$:

(3) $$\begin{array}{l} \ell_{P}\left(\theta |x\right)=\sum_{i}\sum_{j}\log\left\{\sum_{a_{i}}\sum_{a_{j}}\mathrm{Pr}{\left(K_{\mathrm{ij}}=\mathrm{PO}/\mathrm{OP}|z_{i},z_{j},a_{i},a_{j}\right)}^{\omega_{\mathrm{ij}}}\times \right.\\ \left.{\left(1-\mathrm{Pr}\left(K_{\mathrm{ij}}=\mathrm{PO}/\mathrm{OP}|z_{i},z_{j},a_{i},a_{j}\right)\right)}^{1-\omega_{\mathrm{ij}}}\times f(a_{i}|l_{i}^{*})f(a_{j}|l_{j}^{*})\right\}. \end{array}$$

We will now specify the two main elements of Equation (3), namely the probability of observing the PO/OP kinship, and the probability density of age given length.

#### 2.2.1 Probability of kinship

We modelled the female and male adult abundance separately; thus, for every PO/OP comparison between two individuals we had to consider, conditional on the sexes, both combinations of which individual in older and thus the potential parent. We will first present the formulae for the simple species, followed by those for the complex species. The probability of any comparison between i and j being PO/OP is the same as the sum of testing for PO and OP separately, thus we only present the PO probabilities. For the simple species this became

(4) $$\begin{array}{l} \mathrm{Pr}\left(K_{\mathrm{ij}}=\mathrm{MO}|z_{i},z_{j},a_{i},a_{j}\right)=\\ \;I\left(y_{i}+\alpha_{♀}\leq y_{j}\right)\times {\left(N_{♀,y_{j}}\right)}^{-1}\times \left(\begin{array}{ll} 1; & \text{if}\;c_{i}\geq y_{j}\\ \phi_{i}\left(c_{i},y_{j}\right); & \text{if}\;c_{i}<y_{j} \end{array}\right\} \end{array}$$

for the females, and

(5) $$\begin{array}{l} \mathrm{Pr}\left(K_{\mathrm{ij}}=\mathrm{FO}|z_{i},z_{j},a_{i},a_{j}\right)=\\ \;I\left(y_{i}+\alpha_{♂}\leq y_{j}\right)\times {\left(N_{♂,y_{j}}\right)}^{-1}\times \left(\begin{array}{ll} 1; & \text{if}\;c_{i}\geq y_{j}\\ \phi_{i}\left(c_{i},y_{j}\right); & \text{if}\;c_{i}<y_{j} \end{array}\right\} \end{array}$$

for the males. Here, I is an indicator function that returns 1 if its argument is true and 0 otherwise, MO and FO refer to mother‐offspring and father‐offspring, respectively, y denotes the birth year, α the age of maturity, $N_{s,t}$ the total adult abundance of sex s in year t, c the year of capture, and $\phi_{i}\left(t_{1},t_{2}\right)$ the survival function for individual i from $t_{1}$ to $t_{2}$. As survival was assumed constant, $\phi_{i}\left(t_{1},t_{2}\right)$ was defined as $\phi^{t_{2}-t_{1}}$. Even though females could only have one litter whereas males could father multiple litters, their ERROs were formulated similarly, that is, the reciprocal of the total mature abundance of their respective sexes. For the complex species, the probability of an MO pair thus became

(6) $$\begin{array}{l} \mathrm{Pr}\left(K_{\mathrm{ij}}=\mathrm{MO}|z_{i},z_{j},a_{i},a_{j}\right)=\\ \;I\left(y_{i}+\alpha_{♀}\leq y_{j}-1\right)\times {\left(N_{♀,y_{j}-1}\times \phi \right)}^{-1}\times \left(\begin{array}{ll} 1; & \text{if}\;c_{i}\geq y_{j}\\ \phi_{i}\left(c_{i},y_{j}\right); & \text{if}\;c_{i}<y_{j} \end{array}\right\} \end{array}$$

and the probability of an FO pair became

(7) $$\begin{array}{l} \mathrm{Pr}\left(K_{\mathrm{ij}}=\mathrm{FO}|z_{i},z_{j},a_{i},a_{j}\right)=\\ \;I\left(y_{i}+\alpha_{♂}\leq y_{j}-1\right)\times {\left(N_{♂,y_{j}-1}\right)}^{-1}\times \left(\begin{array}{ll} 1; & \text{if}\;c_{i}\geq y_{j}-1\\ \phi_{i}\left(c_{i},y_{j}-1\right); & \text{if}\;c_{i}<y_{j}-1 \end{array}\right\}. \end{array}$$

The two key differences between the complex species relative to the simple one were that (1) a potential father only needed to have been alive the year before the birth of the offspring, whereas a potential mother needed to have survived until birthing, and (2) the potential parents needed to have matured at least 1 year before the birth year. To illustrate this, imagine that we are comparing two individuals from the complex species, where the parent is female, and we know the individuals' ages. The offspring was caught in year 50 at age 3, and thus born in year 47. The potential parent was female, and caught in year 45 and would have needed to survive for at least 2 years in order to be a potential parent; she was 36 years old at the time of capture, and thus born in year 9. The ERRO for this parent in the year of mating, that is, the year before the birth year of *j*, is the reciprocal of the number of females alive in that year who also survived 1 year of gestation, which is ϕ. Therefore, the probability that i is the mother of j would be:

(8) $$\begin{array}{l} \mathrm{Pr}\left(K_{\mathrm{ij}}=\mathrm{MO}|z_{i},z_{j},a_{i},a_{j}\right)=I\left(9+19\leq 47-1\right)\times {\left(N_{♀,y_{j}-1}\times \phi \right)}^{-1}\times \phi^{47-45}\\ =1\times {\left(N_{♀,y_{j}-1}\times \phi \right)}^{-1}\times \phi^{2}\\ =\phi {\left(N_{♀,y_{j}-1}\right)}^{-1} \end{array}$$

Every comparison, given $a_{i}$ and $a_{j}$, contains a signal about the adult population in a specific year. We assumed a constant population size, and thus $N_{s,t}=N_{s}$. We also developed and tested a model that included sex‐specific growth parameters. This model was internally inconsistent and therefore not included in the main body of this manuscript for any formal inference. However, we did include the derivations and some results in Appendix C.

#### 2.2.2 Probability density of age given length

We had an assumed true length‐at‐age curve $l\left(a\right)$ (Equation (1)) and we knew that there was measurement error on lengths. Denoting the *measured* length as $l^{*}$, we derived the probability density $f\left(a|l^{*}\right)$ using Bayes' rule as follows:

(9) $$f\left(a|l^{*}\right)=f\left(l^{*}|a\right)f\left(a\right)f{\left(l^{*}\right)}^{-1}.$$

Measured length given age $l^{*}|a$ was assumed to follow a discretised Normal distribution, as lengths were rounded to the nearest centimetre. We followed Roy (2003) in defining this distribution as

(10) $$f\left(l^{*}|a\right)=\Phi \left(\frac{l^{*}-\mu -0.5}{\sigma_{l}}\right)-\Phi \left(\frac{l^{*}-\mu +0.5}{\sigma_{l}}\right),$$

where Φ denotes the standard normal cumulative distribution function, the expectation μ is given by Equation (1), and $\sigma_{l}$ captures the standard deviation of length measurement error. As the sampling probability in the simulation was unrelated to age, the age distribution of sampled individuals was the same as the age distribution in the whole population, and we did not need to distinguish between the two. We assumed that the population had a stable age distribution with no growth, which meant that the distribution of ages, had we not imposed a maximum age, would have been geometric with shape parameter being equal to the mortality rate, which is 1−ϕ. Acknowledging that there was a maximum age, $a_{\max}$, we needed to condition on the age being at most this age, and thus

(11) $$f\left(a\right)=\left(\begin{array}{ll} \frac{{\left(\phi \right)}^{a}\left(1-\phi \right)}{1-{\left(\phi \right)}^{a_{\max}+1}}; & \text{if}\;0\leq a\leq a_{\max}\\ 0; & \text{otherwise} \end{array}\right.,$$

where the numerator and denominator were the geometric probability mass and cumulative distribution functions, respectively. Note here that we used the definition of a geometrically distributed variable being the number of failures (survival) until a success (death) occurs. Finally, the probability density function on measured length became

(12) $$f\left(l^{*}\right)=\sum_{a=0}^{a_{\max}}f\left(l^{*}|a\right)f\left(a\right).$$

### 2.3 Fitting

**FIGURE 2:**
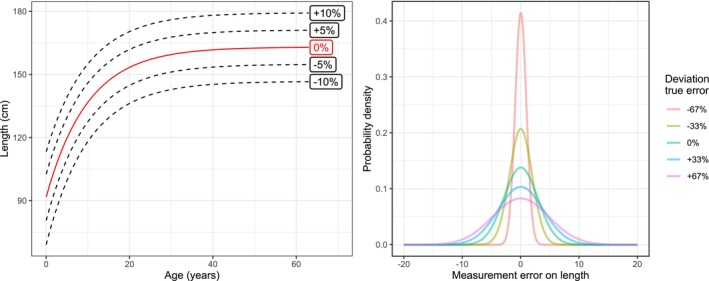
The left panel shows the five growth curves that were used in the scenarios tested in this study. The true growth curve is indicated in red; the black dotted lines show the incorrect ones, which were constructed by shifting the growth curve up and down in steps of 5%. These shifts were aimed to represent real variation in growth curves between populations of the same shark species (Bradley, Conklin, Papastamatiou, McCauley, Pollock, Kendall, et al., 2017). The right panel shows five measurement errors used in this study. The true simulated error was 2.89 cm, and the other measurement errors were chosen by deviating from this error in both directions.

The parameters in the CKMR model were estimated from the samples collected in the last two years of the simulation by maximising the pseudo‐log‐likelihood, which can involve prohibitively long computation time. To resolve this, we restricted the number of pairwise comparisons. Many pairwise comparisons resulted in identical probabilistic statements, and thus in practice only needed to be derived once. As we considered adult abundance for both sexes separately, we estimated two parameters: $N_{♀}$ and $N_{♂}$. All other parameters, such as ϕ, were assumed known and fixed. To each of the 2000 population realisations (1000 for each species) we fitted the appropriate POP model with varying degrees of length measurement error and growth curves, which was achieved by altering some of the fixed parameters. Specifically, we assumed five different standard deviations for length measurement error: the correct one, a 33% and 67% underestimate, and a 33% and 67% overestimate. We also considered five different growth curves: the correct one, two that were shifted upwards by 5% and 10%, and two that were shifted downwards by 5% and 10%. These growth curve shifts were aimed to represent real variation in growth curves between populations of the same shark species (Bradley, Conklin, Papastamatiou, McCauley, Pollock, Kendall, et al., 2017). This resulted in a total of 25 combinations or ‘scenarios’. We labelled these scenarios using the format ‘ME±XX:GC ± YY’, where ME refers to the measurement error, XX denotes the percentage over‐ or underestimate, GC stands for growth curve, and YY denotes the percentage of up‐ or downwards shifting; for example, the scenario with a 33% overestimated standard deviation of length measurement error and a 5% downshifted growth curve had the label ME+33:GC‐5. These measurement errors and growth curves are visualised in more detail in Figure 2. Considering 25 scenarios for every simulation resulted in the fitting of 50,000 models in total. To keep computation time to a minimum, we implemented most of the fitting process in C++.

### 2.4 Variance and performance

To evaluate the performance of the estimator, we present the following metrics: (i) mean error and mean relative error to evaluate a potential bias; (ii) median error and median relative error to evaluate the median bias, which uses the median instead of the mean, as the median is often more appropriate when distributions are skewed. In addition, the mean absolute error (MAE) and root mean square error (RMSE) are presented in supplemental tables. The definitions of the six metrics are given in Appendix A.1. Furthermore, we derived the 95% log‐normal confidence interval (CI) coverage to evaluate the performance of these CIs in the correct growth curve scenarios. Variance was estimated from the Hessian matrix produced by the maximum likelihood estimation, and averaged over these 1000 estimated standard errors. We can treat the pseudo‐likelihood as a true likelihood as long as sampling was sparse (see Section 2.2). It is unclear if this criterion was met in our study, as we took 750 samples from a population with roughly 8500 individuals. If sampling is not sparse, the estimated variance could be negatively biased as the pairwise comparisons are not approximately independent. To explore the extent of this potential bias, we evaluated how well the average estimated standard error estimated the empirical standard deviation of population estimate errors across the 1000 simulations for each species. We include definitions of these in Appendix B.1.

## 3 RESULTS

The mean number of POPs for all sampling realisations was 48.6 (range: 25–76) for the simple species and 55.6 (range: 31–90) for the complex species. Mean simulated adult abundances in the final year of the simulation were 794 and 793 (range: 630–992 and 600–1019; ♀ and ♂) for the simple species and 514 and 650 (range: 400–683 and 516–824; ♀ and ♂) for the complex species. A small number of recaptures between the 2years of sampling, that is, that some individuals were sampled at more than one sampling event, occurred in every simulation. This ranged from 4 to 25 recaptured individuals for the simple species and 5–30 individuals for the complex species. The simulated mean annual growth rate was 0.999 for both sexes of the simple species, and 1.001 and 0.998 for the males and females of the complex species, respectively; the mean annual growth for any simulation was always within 0.3 percent point from the mean across all simulations. The fitting algorithm did not always converge when the measurement error standard deviation and/or the growth curve was (very) negatively biased. Whenever this happened, it happened for most of the simulations in that scenario. Therefore, we excluded the scenarios where this happened from the analysis, which led to the exclusion of scenarios ME‐67:GC‐10, ME‐67:GC‐5, ME‐67:GC + 0, ME‐33:GC‐10, ME‐33:GC‐5, and ME+0:GC‐10. In the other scenarios, all models converged successfully.

**FIGURE 3:**
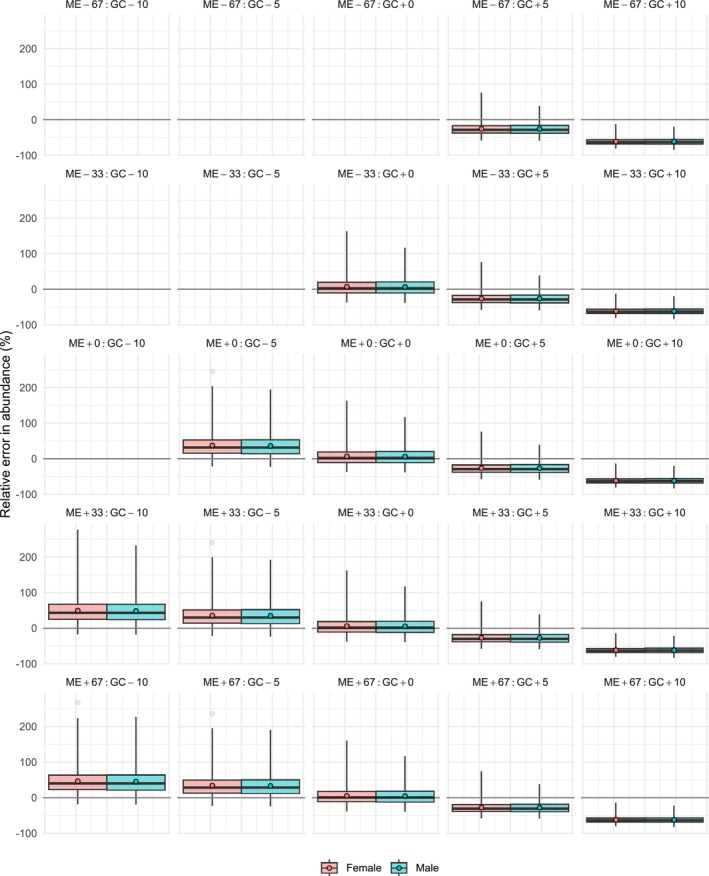
Box plots for the error in estimated sex‐specific adult abundance relative to the true abundance for the simple species. We only present the results for scenarios in which the optimiser consistently converged; this meant that some scenarios were left blank. Box plots show the interquartile range (IQR) and the median; the mean is indicated by the darker filled circle; the vertical lines cover five times the IQR; and all values outside of that are indicated as outliers. The scenarios were labelled using the format ‘ME±XX:GC ± YY’, where ME refers to the measurement error, XX denotes the percentage over‐ or underestimate, GC stands for growth curve, and YY denotes the percentage of up‐ or downwards shifting; for example, the scenario with a 33% overestimated standard deviation of length measurement error and a 5% downshifted growth curve had label ME+33:GC‐5.

**FIGURE 4:**
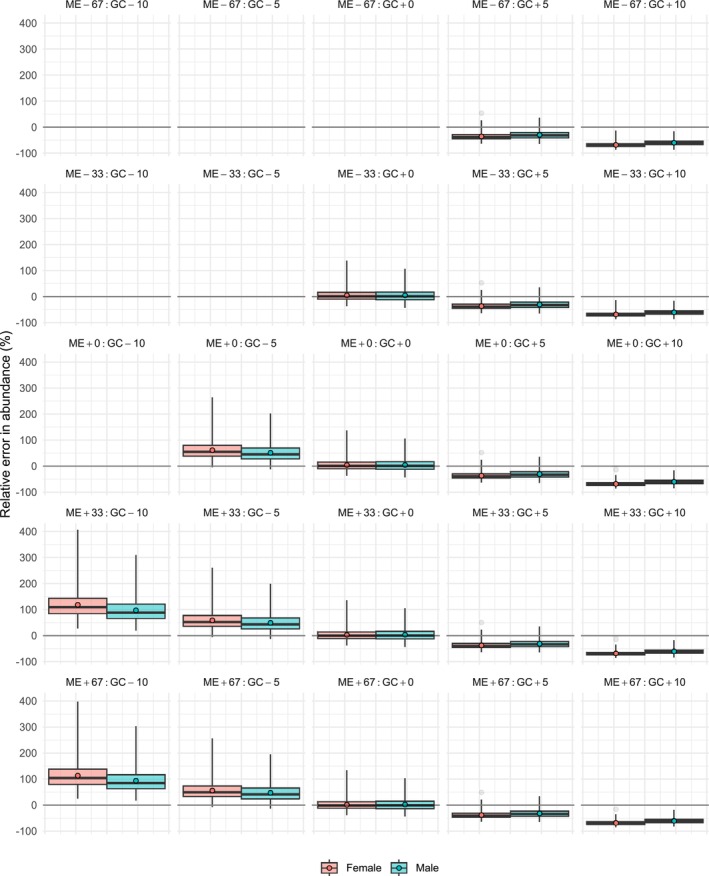
Box plots for the error in estimated sex‐specific adult abundance relative to the true abundance for the complex species. We only present the results for scenarios in which the optimiser consistently converged; this meant that some scenarios were left blank. Box plots show the interquartile range (IQR) and the median; the mean is indicated by the darker filled circle; the vertical lines cover five times the IQR; and all values outside of that are indicated as outliers. The scenarios were labelled using the format ‘ME±XX:GC ± YY’, where ME refers to the measurement error, XX denotes the percentage over‐ or underestimate, GC stands for growth curve, and YY denotes the percentage of up‐ or downwards shifting; for example, the scenario with a 33% overestimated standard deviation of length measurement error and a 5% downshifted growth curve had label ME+33:GC‐5.

For the simple species, median errors for ${\hat{N}}_{s}$ when using correct measurement error and growth curve specification (ME+0:GC + 0) were 20.83 and 22.52 (relative: 2.57% and 2.79%; ♀ and ♂) individuals (Figure 3; Tables A1 and A2). For the complex species, median errors for ${\hat{N}}_{s}$ when using correct measurement error and growth curve specification were 6.48 and 10.28 (relative: 1.35% and 1.59%; ♀ and ♂) individuals (Figure 4; Tables A3 and A4). For the simple species, median relative errors in abundance estimates were positive but close to zero for all deviations from the true standard deviation of length measurement error provided that the growth curve was correctly specified, although they were slightly larger for the females (Figure 3, also Table A1). For any given measurement error standard deviation, we observed a trend from a positive median error to a negative median error as we shifted the growth curve upwards (Figures 3 and 4). When growth curves were shifted down 5%, this resulted in median relative errors of around 30% for the simple species, and between 30% and 60% for the complex species. Shifting growth curves up by 5% resulted in median relative errors of −30% for the simple species, and between −30% and −40% for the complex species.

When the growth curve was correctly specified, the 95% CI coverage (rounded to one decimal place) for the simple species adult abundance estimates ranged from 96.1% to 96.4%, and ranged from 94.4% to 95.9% for the complex species estimates (Table B3). For a given growth curve, no relation between the measurement error standard deviation and CI coverage became apparent. Incorrectly specified growth curves severely lowered the CI coverage for all measurement errors for both species (Table B3). When the growth curve was correctly specified, the empirical standard errors ranged from 180.97 to 182.56 for the male simple species, and 190.85 to 192.56 for the female simple species (Table B1); for the complex species, these errors ranged from 145.77 to 148.42 for the males, and from 107.51 to 109.27 for the females (Table B2). Given a growth curve, increasing the measurement error standard deviation seemed slightly decrease the empirical standard errors, for all species and sexes. Given an assumed standard deviation of measurement error, the empirical standard errors decreased as the growth curve was shifted upwards. When the growth curve was correctly specified, the average estimated standard errors ranged from 181.04 to 183.04 for the male simple species, and 183.54 to 185.51 for the female simple species (Table B1); for the complex species, these errors ranged from 139.79 to 142.41 for the males, and from 102.99 to 105.71 for the females (Table B2). The changes in average estimated standard errors between the scenarios follow a similar pattern to the empirical standard errors.

Whenever the growth curve was correctly specified, average model estimated standard errors were always (slightly) lower than empirical standard errors in all scenarios and both species, except for the male simple species (Tables B1 and B2). The underestimation of the empirical standard error by the average estimated standard error was always <5%. Deviations from the correct growth curve increased underestimation in all cases (Tables B1 and B2).

## 4 DISCUSSION

In this study, we explored the effects of incorrect age inference from length measurements on CKMR estimates of adult abundance through misspecifying the length measurement error and the growth curve in various ways. The number of POPs discovered in our simulation was in the vicinity of the 50–100 kin pairs recommended for a CKMR application (Bravington, Skaug, & Anderson, 2016), albeit on the lower end. Overall, an incorrect assumed standard deviation of measurement error mostly impacted the convergence likelihood of the fitting algorithm, whenever this standard deviation was assumed to be smaller in the fitting than was true for the simulation. Whenever the measurement error standard deviation was high enough to allow for convergence, it made little difference whether it was the true value or if a much higher standard deviation was assumed. This would suggest that if researchers are ever unsure about whether their assumed degree of spread in length measurement error is correct, it is safer to overestimate it. A misspecified growth curve, on the other hand, had drastic effects on the estimation of all parameters: a 5% shift away from the true growth curve resulted in biases ranging from −60% to +40%; estimated and empirical standard errors seemed to scale with the abundance estimates, and shifting away from the true growth curve resulted in an increased underestimation of empirical standard errors.

The model performed well under correct specification (scenario ME+0:GC + 0), although the positive median relative error in adult abundance estimates suggests a positive median bias. This error was more extreme for the simple than for the complex species. A bias in the estimates is not uncommon for maximum likelihood methods when the sample size is small, which could be true in our study as the number of sampled POPs never surpassed 76 for the simple species and 90 for the complex species. However, it could also be that this shows a slight positive bias in the method itself, especially as a previous CKMR simulation study by Conn et al. (2020) found small positive biases in the abundance estimates, too. We can express the empirical standard errors for the correct scenarios as percentages of the associated mean simulated abundances in the final year. This gives standard errors relative to the mean (also known as coefficients of variation) of 23.0% and 24.3% for the male and female estimates of the simple species, respectively, and 22.7% and 21.2% for the male and female estimates of the complex species, respectively. These are high but not uncommon for real‐life population studies. The 95% log‐normal confidence intervals (CIs) seemed to accurately represent the uncertainty around the estimates whenever the correct growth curve specification was used, as the coverage ranged from 94.4% to 96.4%. Nonetheless, the coverage always exceeded 95% when the entire model was correctly specified, which could indicate that the 95% log‐normal CIs were slightly conservative.

In this study, we assumed that all individual sharks followed the specified growth curve perfectly, and any variation in lengths for a given age resulted from measurement error. This is a simplification of reality, and future research could focus on ways to accommodate natural variation in length at a given age, which could be a function of age in itself. As an incorrect standard deviation of length measurement error seemed to have little effect on point estimates, we believe that, when in doubt, it is preferable to assume a higher standard deviation as this improves how likely it is that the fitting algorithm converges.

The effects of deviating from the true growth curve on the adult abundance estimates were substantial. When growth curves were shifted by 5% we often observed median relative errors of over 30%. This strongly highlights the sensitivity of the method to correct age estimation. Empirical standard errors were also increasingly underestimated when growth curves were shifted away from the truth. This effect was stronger when the growth curve was shifted upwards, that is, when ages were being underestimated. An underestimation of uncertainty could be a consequence of the comparisons not being truly independent, that is, a violation of the sparse sampling assumption. It is important here to note that we did not evaluate the standard deviation of abundance estimates but rather of the error in abundance estimates (see Appendix B.1). The true abundance was different for every simulation, so we could not use the standard deviation of the abundance estimates itself, since this would partly capture the stochasticity of the simulation process. To overcome this, we used the standard deviation of the error in abundance estimates, which should be a more robust measure of the true standard error. This should not be a problem as long as estimation is unbiased; however, our results indicate a slight positive bias, which could have impacted the accurateness of the empirical standard error in being a measure of true standard error. CI coverage was most severely impacted by incorrect growth curves; however, this was likely mostly due to the bias in the estimates in those scenarios. In real‐world applications, researchers could potentially check the correctness of their assumed growth curve by assessing the distribution of lengths/ages among the sampled individuals. If many of the observed lengths are either associated with very low ages or are close to asymptote, or in some other way exhibit an unexpected sampled age distribution given the sampling scheme, then this could be an indication that the assumed growth curve is incorrect (or that sampling assumptions are violated).

Even though recaptures should be rare (as long as sampling is sparse), they did occur in our simulations between sampling years. These recaptures did not pose any problems within the analysis, for example, getting mistaken for a different genetic relationship, and thus we retained the recaptures in our data. Alternatively, duplicate samples can be excluded from the analysis when there is reason to do so. We hypothesise that excluding recaptures would likely increase estimates of uncertainty, as fewer observations are used for the analysis. We are unaware of any study that investigated the extent to which including recaptures could potentially affect precision or even bias in CKMR estimation, and we believe that this could be a great topic for future research. Whenever it is known that multiple samples belong to a single individual, there exists the potential for extending CKMR by incorporating some form of capture‐recapture into the method (Bravington, Skaug, & Anderson, 2016; Otis et al., 1978). Additionally, it could also allow us to fit the growth curve jointly with the CKMR model, instead of assuming it to be known by extrapolating from other studies (Bravington et al., 2019). This could create a situation where one collects new samples every year to update the model, thereby continuously improving the estimates not only of the abundance and trend, but also of the growth curve: in a Bayesian framework, one could use the initial growth curve as prior information, and then update the posterior every year as more information is collected.

In our model, we did not allow for any growth or decline in the population size over time. Our simulated populations exhibited no systematic growth, but the stochastic nature of the process did lead to some random growth/decline. One could consider estimating a growth rate, or assume a small range of growth rates (see Hillary et al. (2018) for an example with white sharks (*Carcharodon carcharias*)). The main challenge would be to understand how including a growth rate parameter affects the assumed age distribution $f\left(a\right)$. We can imagine three general population growth scenarios. If a population is stable but growing or in decline, the assumed age distribution will be geometric and depend on a combination of survival and growth rate (Caswell, 2006, Section 4.5.2.1). The second scenario is when a population exhibits changing growth or decline, in which case there is no stable age structure. We believe that this scenario is intractable, and it would make a good subject for a robustness study to see how much it affects estimation. The third scenario would be where there is no expected population growth or decline but there is demographic stochasticity, which in practice could result in deviations from the stable age structure. For this scenario, an option could be to use the method described by Hillary et al. (2018), where the measured lengths were binned and a multinomial distribution was fit to these binned data to estimate the distribution of sampled ages. Still, this could be a topic for future research to see what other methods exist to find the distribution of (sampled) ages.

CKMR involves many pairwise comparisons, which often involve many identical probabilistic statements. To limit computation time, we evaluated unique probabilistic statements only once. If further computational improvements are required, it is possible to reduce the number of pairwise comparisons that are evaluated by excluding a subset of comparisons from the analysis. For example, the length‐age relationships are often much clearer for younger animals, and therefore one could choose to only consider animals up to a certain size as potential offspring (Trenkel et al., 2022).

In our simulation and model, we assumed some life history traits to be fixed and known, but this is not always required for CKMR. We estimated sex‐specific adult abundance only in our model and assumed quantities such as survival to be known and fixed. In order to relax the assumption of a fixed and known survival parameter ϕ, one could estimate it by including half‐sibling pairs alongside parent‐offspring pairs (Bravington, Skaug, & Anderson, 2016). Parent‐offspring pairs can be used to model fecundity as long as the parameter appears explicitly in the model, which could be the case when fecundity varies with the size or age of animals (Bravington, Skaug, & Anderson, 2016, Section 3.1.4) We are unaware of any attempts to estimate time‐varying fecundity or survival, and we believe this to be a potential direction for future research. Moreover, we assumed maturity to be knife‐edge as it slightly reduces the complexity of the model. However, if maturity occurs more gradually, then this can be accommodated by adding a fecundity curve to the model (e.g., a logistic curve; Conn et al., 2020). We also imposed a fixed and known maximum age in the simulation, mostly to reduce computation time. In reality, animals do not always have a maximum age; in such cases, one could set the maximum age equal to an age that the animal has practically zero probability of reaching. Further, we made the assumption that sampling was random with respect to age, that is, that there is no selectivity, which does not necessarily need to be true in reality. When accounting for ageing error when there is selectivity, it will be necessary to include some function relating true age to observed age, which would depend on the probability of being sampled at a given true age. Finally, we have not considered fishing‐induced mortality, as our case study concerned an area protected from fishing. This and other anthropomorphic sources of mortality should be accounted for whenever they are present, analogously to Bravington, Grewe, and Davies (2016).

When a promising method like CKMR is first presented, one can see the appeal to start studying populations as quickly as possible. Benchmark comparisons could be useful (e.g., Ruzzante et al., 2019) to compare a new method to some ‘truth’. However, these comparisons can be ambiguous when it is unclear how accurate the benchmark truly is. Simulation studies, such as this one (and see Conn et al. (2020) for the effects of unmodelled spatial heterogeneity on CKMR), are a key part of understanding when the CKMR method works well and when it does not. We believe the CKMR method has great potential and, in some cases, is an improvement over other methods, but our study confirms that care that needs to be taken when ageing is biased. In such cases epigenetic ageing could be preferable, even though epigenetic ageing can still involve substantial uncertainty (e.g., Larison et al., 2021; Prado et al., 2021) and relies strongly on the quality of the training data (Mayne et al., 2023).

## AUTHOR CONTRIBUTIONS

**Felix T. Petersma:** Conceptualization (lead); formal analysis (lead); methodology (lead); visualization (lead); writing – original draft (lead); writing – review and editing (equal). **Len Thomas:** Conceptualization (supporting); formal analysis (supporting); methodology (supporting); visualization (supporting); writing – original draft (supporting); writing – review and editing (equal). **Danielle Harris:** Conceptualization (supporting); formal analysis (supporting); methodology (supporting); visualization (supporting); writing – original draft (supporting); writing – review and editing (equal). **Darcy Bradley:** Conceptualization (supporting); formal analysis (supporting); methodology (supporting); writing – review and editing (equal). **Yannis P. Papastamatiou:** Conceptualization (supporting); formal analysis (supporting); methodology (supporting); writing – review and editing (equal).

## CONFLICT OF INTEREST STATEMENT

The authors declare no conflicts of interest.

### OPEN RESEARCH BADGES

This article has earned Open Data and Open Materials badges. Data and materials are available at https://doi.org/10.5281/zenodo.10727211.

## Figures and Tables

**FIGURE C1:**
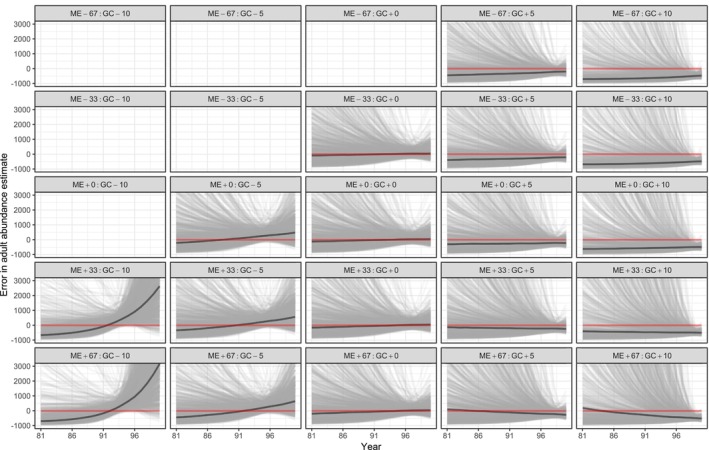
Plots of the 1000 simple female adult population trends for the simple species for the last 20 years of the simulation, for the 19 scenarios that resulted in successful fits. The median of these 1000 trends is indicated in dark grey, and the truth adult abundance is indicated in red. The scenarios were labelled using the format ‘ME±XX:GC ± YY’, where ME refers to the measurement error, XX denotes the percentage over‐ or underestimate, GC stands for growth curve, and YY denotes the percentage of up‐ or downwards shifting; for example, the scenario with a 33% overestimated standard deviation of length measurement error and a 5% downshifted growth curve had label ME+33:GC‐5.

**FIGURE C2:**
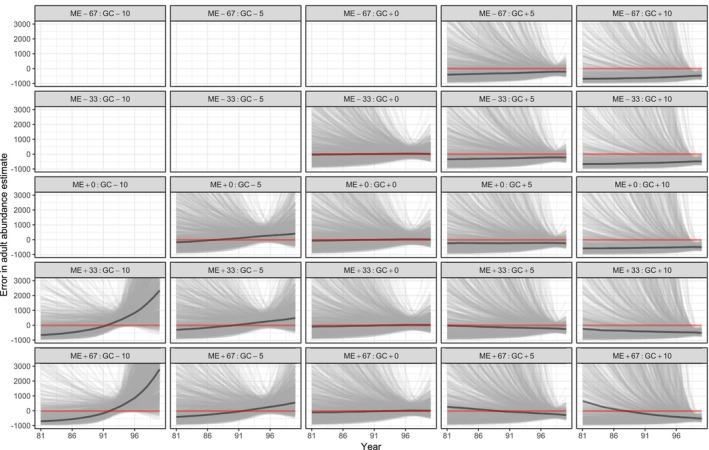
Plots of the 1000 estimated male adult population trends for the simple species for the last 20 years of the simulation, for the 19 scenarios that resulted in successful fits. The median of these 1000 trends is indicated in dark grey, and the truth adult abundance is indicated in red. The scenarios were labelled using the format ‘ME±XX:GC ± YY’, where ME refers to the measurement error, XX denotes the percentage over‐ or underestimate, GC stands for growth curve, and YY denotes the percentage of up‐ or downwards shifting; for example, the scenario with a 33% overestimated standard deviation of length measurement error and a 5% downshifted growth curve had label ME+33:GC‐5.

**FIGURE C3:**
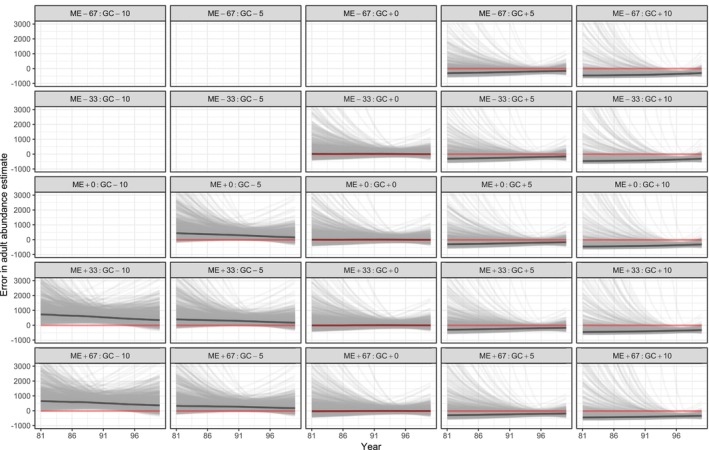
Plots of the 1000 estimated female adult population trends for the complex species for the last 20 years of the simulation, for the 19 scenarios that resulted in successful fits. The median of these 1000 trends is indicated in dark grey, and the truth adult abundance is indicated in red. The scenarios were labelled using the format ‘ME±XX:GC ± YY’, where ME refers to the measurement error, XX denotes the percentage over‐ or underestimate, GC stands for growth curve, and YY denotes the percentage of up‐ or downwards shifting; for example, the scenario with a 33% overestimated standard deviation of length measurement error and a 5% downshifted growth curve had label ME+33:GC‐5.

**FIGURE C4:**
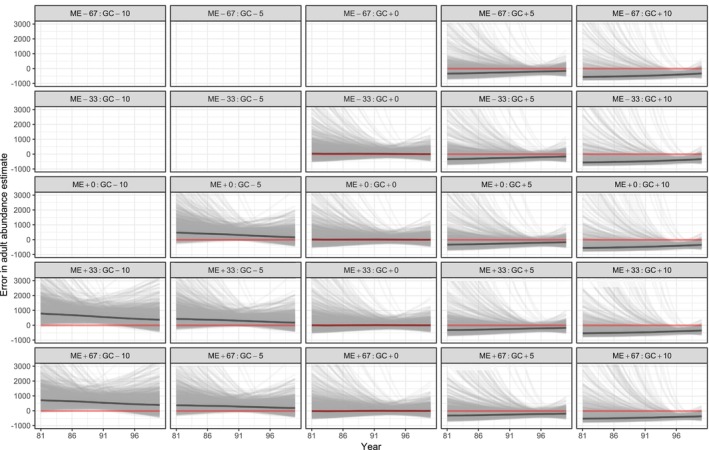
Plots of the 1000 estimated male adult population trends for the complex species for the last 20 years of the simulation, for the 19 scenarios that resulted in successful fits. The median of these 1000 trends is indicated in dark grey, and the truth adult abundance is indicated in red. The scenarios were labelled using the format ‘ME±XX:GC ± YY’, where ME refers to the measurement error, XX denotes the percentage over‐ or underestimate, GC stands for growth curve, and YY denotes the percentage of up‐ or downwards shifting; for example, the scenario with a 33% overestimated standard deviation of length measurement error and a 5% downshifted growth curve had label ME+33:GC‐5.

## Data Availability

The R scripts used for the simulation and analysis in this study are publicly available at https://doi.org/10.5281/zenodo.10727211.

## APPENDIX A PERFORMANCE METRICS

### A.1 Definitions

Every simulation had a different realised sex‐specific adult abundance, due to stochasticity in the demographic processes such as fecundity. Therefore, the error in the abundance estimate for a specific simulation was defined as the estimated abundance minus the true abundance for said simulation, such that

(A1)
$$\varepsilon_{s,m}={\hat{N}}_{s}^{\left(m\right)}-N_{s,100}^{\left(m\right)},$$

where m∈1,…,M denotes the simulation index and $s\in \left\{♀,♂\right\}$ denotes the sex. We define the relative error as relative to the true adult abundance in year 100 of the simulation, that is,

$$\frac{\varepsilon_{s,m}}{N_{s,100}^{\left(m\right)}}.$$

The mean error, median error, mean relative error, median relative error, and the mean absolute error all easily follow from this, where ‘mean’ always refers to the arithmetic mean. Finally, the root mean square error (RMSE) is defined as

$$\text{RMSE}=\sqrt{\frac{1}{M}\sum_{m=1}^{M}\varepsilon_{s,m}^{2}}.$$

All of these definitions match the definitions common in other literature.

### A.2 Results

**TABLE A1:** Performance metrics for the estimation of parameter $N_{♀}$, extracted from 1000 simulations of the simple shark population.

Scenario	Mdn. Rel. Err	Mean Rel. Err.	Mdn. Err.	Mean err.	MAE	RMSE
ME‐67:GC + 5	−28.48	−25.66	−223.83	−203.94	220.61	245.14
ME‐67:GC + 10	−62.69	−61.36	−495.14	−487.72	487.72	495.50
ME‐33:GC + 0	2.88	7.32	22.72	58.23	142.91	201.11
ME‐33:GC + 5	−28.54	−25.71	−224.94	−204.40	220.93	245.38
ME‐33:GC + 10	−62.78	−61.34	−495.26	−487.58	487.58	495.28
ME+0:GC‐5	31.80	37.56	252.60	298.59	304.39	390.33
ME+0:GC + 0	2.57	7.14	20.83	56.78	142.47	200.48
ME+0:GC + 5	−28.72	−25.85	−227.03	−205.50	221.78	246.05
ME+0:GC + 10	−62.70	−61.23	−494.06	−486.74	486.74	494.33
ME+33:GC‐10	43.85	50.21	343.32	399.15	400.98	488.70
ME+33:GC‐5	30.59	36.34	243.51	288.88	295.53	381.69
ME+33:GC + 0	2.23	6.76	18.13	53.74	141.65	199.10
ME+33:GC + 5	−29.21	−26.29	−230.69	−209.01	224.53	248.47
ME+33:GC + 10	−62.81	−61.25	−494.00	−486.85	486.85	494.26
ME+67:GC‐10	41.04	47.21	320.66	375.24	377.73	466.13
ME+67:GC‐5	29.16	34.90	232.41	277.39	285.22	371.65
ME+67:GC + 0	1.79	6.12	13.53	48.64	140.45	196.86
ME+67:GC + 5	−29.80	−26.97	−235.30	−214.35	228.79	252.27
ME+67:GC + 10	−62.85	−61.37	−495.30	−487.84	487.84	495.04

*Note*: The columns are, from left to right: scenario, median relative error, mean relative error, median error, mean error, mean absolute error, and root mean square error. Scenarios ME‐67:GC‐10, ME‐67:GC‐5, ME‐67:GC + 0, ME‐33:GC‐10, ME‐33:GC‐5, and ME+0:GC‐10 were not included as (most of) the simulations did not fit successfully. Scenario 3‐3 uses the correct measurement error (2.89) and growth curve specification.

**TABLE A2:** Performance metrics for the estimation of parameter $N_{♂}$, extracted from 1000 simulations for the simple shark population.

Scenario	Mdn. Rel. Err	Mean Rel. Err.	Mdn. Err.	Mean err.	MAE	RMSE
ME‐67:GC + 5	−28.40	−25.93	−223.56	−206.14	217.13	243.78
ME‐67:GC + 10	−62.28	−61.38	−488.98	−487.42	487.42	494.97
ME‐33:GC + 0	2.96	6.74	24.43	52.92	144.84	189.98
ME‐33:GC + 5	−28.53	−25.99	−224.78	−206.64	217.45	244.05
ME‐33:GC + 10	−62.41	−61.37	−489.77	−487.34	487.34	494.79
ME+0:GC‐5	31.59	36.63	247.66	289.87	297.53	374.35
ME+0:GC + 0	2.79	6.55	22.52	51.44	144.49	189.38
ME+0:GC + 5	−28.72	−26.14	−226.29	−207.83	218.33	244.83
ME+0:GC + 10	−62.30	−61.28	−489.39	−486.58	486.58	493.89
ME+33:GC‐10	43.72	49.00	341.71	387.75	390.42	470.34
ME+33:GC‐5	30.62	35.41	238.47	280.12	289.00	365.82
ME+33:GC + 0	2.34	6.16	19.50	48.30	143.76	188.06
ME+33:GC + 5	−29.16	−26.59	−228.60	−211.41	221.07	247.38
ME+33:GC + 10	−62.32	−61.30	−489.56	−486.78	486.78	493.90
ME+67:GC‐10	40.98	46.03	320.23	364.13	367.87	448.20
ME+67:GC‐5	29.36	33.96	229.31	268.62	279.08	355.90
ME+67:GC + 0	1.72	5.51	13.73	43.13	142.59	185.95
ME+67:GC + 5	−29.89	−27.27	−233.68	−216.81	225.38	251.36
ME+67:GC + 10	−62.58	−61.44	−490.18	−487.86	487.86	494.77

*Note*: The columns are, from left to right: scenario, median relative error, mean relative error, median error, mean error, mean absolute error, and root mean square error. Scenarios ME‐67:GC‐10, ME‐67:GC‐5, ME‐67:GC + 0, ME‐33:GC‐10, ME‐33:GC‐5, and ME+0:GC‐10 were not included as (most of) the simulations did not fit successfully. Scenario 3‐3 used the correct measurement error (2.89) and growth curve specification.

**TABLE A3:** Performance metrics for the estimation of parameter $N_{♀}$, extracted from 1000 simulations of the complex shark population.

Scenario	Mdn. Rel. Err	Mean Rel. Err.	Mdn. Err.	Mean err.	MAE	RMSE
ME‐67:GC + 5	−38.21	−35.96	−194.00	−185.43	187.72	199.33
ME‐67:GC + 10	−69.41	−68.34	−355.48	−352.09	352.09	356.40
ME‐33:GC + 0	1.89	5.30	9.12	26.84	82.06	112.47
ME‐33:GC + 5	−38.37	−36.07	−194.96	−185.99	188.23	199.71
ME‐33:GC + 10	−69.34	−68.26	−354.98	−351.70	351.70	355.94
ME+0:GC‐5	55.54	61.97	282.61	318.31	318.38	365.22
ME+0:GC + 0	1.35	4.64	6.48	23.44	81.55	111.32
ME+0:GC + 5	−38.63	−36.31	−195.61	−187.25	189.38	200.68
ME+0:GC + 10	−69.19	−68.16	−353.91	−351.15	351.15	355.29
ME+33:GC‐10	109.97	119.20	556.22	612.37	612.37	666.21
ME+33:GC‐5	53.20	59.79	270.34	307.08	307.24	354.45
ME+33:GC + 0	0.57	3.70	2.63	18.60	80.97	109.82
ME+33:GC + 5	−39.05	−36.72	−197.27	−189.35	191.32	202.37
ME+33:GC + 10	−69.23	−68.08	−353.09	−350.72	350.72	354.71
ME+67:GC‐10	105.31	114.40	534.45	587.65	587.65	641.11
ME+67:GC‐5	50.39	57.13	258.11	293.33	293.59	341.34
ME+67:GC + 0	−0.63	2.49	−3.03	12.36	80.40	108.17
ME+67:GC + 5	−39.51	−37.25	−200.26	−192.09	193.91	204.65
ME+67:GC + 10	−69.15	−68.02	−352.44	−350.42	350.42	354.27

*Note*: The columns are, from left to right: scenario, median relative error, mean relative error, median error, mean error, mean absolute error, and root mean square error. Scenarios ME‐67:GC‐10, ME‐67:GC‐5, ME‐67:GC + 0, ME‐33:GC‐10, ME‐33:GC‐5, and ME+0:GC‐10 were not included as (most of) the simulations did not fit successfully. Scenario 3‐3 used the correct measurement error (2.89) and growth curve specification.

**TABLE A4:** Performance metrics for the estimation of parameter $N_{♂}$, extracted from 1000 simulations for the complex shark population.

Scenario	Mdn. Rel. Err	Mean Rel. Err.	Mdn. Err.	Mean err.	MAE	RMSE
ME‐67:GC + 5	−31.74	−29.79	−203.73	−194.20	201.00	220.13
ME‐67:GC + 10	−61.34	−60.03	−393.89	−390.66	390.66	397.78
ME‐33:GC + 0	2.13	5.35	12.74	34.22	113.74	152.24
ME‐33:GC + 5	−31.95	−29.89	−204.63	−194.80	201.48	220.51
ME‐33:GC + 10	−61.30	−59.99	−393.09	−390.44	390.44	397.48
ME+0:GC‐5	45.75	51.88	297.86	336.61	337.30	401.99
ME+0:GC + 0	1.59	4.91	10.28	31.35	113.06	151.03
ME+0:GC + 5	−32.33	−30.13	−206.50	−196.36	202.81	221.63
ME+0:GC + 10	−61.23	−59.98	−392.55	−390.36	390.36	397.27
ME+33:GC‐10	88.97	98.09	579.49	636.75	636.75	703.85
ME+33:GC‐5	44.11	50.38	288.00	326.83	327.73	392.72
ME+33:GC + 0	1.05	4.24	6.59	27.01	112.18	149.32
ME+33:GC + 5	−32.61	−30.56	−208.75	−199.14	205.21	223.70
ME+33:GC + 10	−61.30	−60.04	−392.71	−390.76	390.76	397.51
ME+67:GC‐10	85.75	94.83	559.04	615.54	615.54	682.57
ME+67:GC‐5	42.24	48.54	276.01	314.85	316.10	381.44
ME+67:GC + 0	0.14	3.35	0.95	21.20	111.13	147.24
ME+67:GC + 5	−33.16	−31.13	−211.30	−202.85	208.45	226.55
ME+67:GC + 10	−61.38	−60.19	−394.36	−391.68	391.68	398.23

*Note*: The columns are, from left to right: scenario, median relative error, mean relative error, median error, mean error, mean absolute error, and root mean square error. Scenarios ME‐67:GC‐10, ME‐67:GC‐5, ME‐67:GC + 0, ME‐33:GC‐10, ME‐33:GC‐5, and ME+0:GC‐10 were not included as (most of) the simulations did not fit successfully. Scenario 3‐3 used the correct measurement error (2.89) and growth curve specification.

## APPENDIX B VARIANCE ESTIMATES

### B.1 Definitions

We used the standard deviation of the error term, that is, standard error, as a measure of uncertainty for estimated adult abundance. We did not use standard deviation of the adult abundance estimates themselves, as these contain stochasticity from the simulation process (every simulation had a slightly different true adult abundance in the final year). We define the simulation‐based or empirical standard error of the adult abundance estimate as

(B1)
$$\sigma_{s}^{N}=\sqrt{\frac{1}{M}\sum_{m=1}^{M}{\left(\varepsilon_{s,m}-{\bar{\varepsilon }}_{s,m}\right)}^{2}},$$

where m∈1,…,M denotes the simulation index, $s\in \left\{♀,♂\right\}$ denotes the sex, and ${\bar{\varepsilon }}_{s,m}$ is the arithmetic mean of $\varepsilon_{s,m}$ (see Equation (A.1)).

We define the model‐based or estimated standard error for a single simulation m as

(B2)
$${\hat{\sigma }}_{s,m}^{N}=\sqrt{\hat{\mathrm{Var}}\left({\hat{N}}_{s}^{\left(m\right)}\right)},$$

where $\hat{\mathrm{Var}}\left({\hat{N}}_{s}^{\left(m\right)}\right)$ is the estimated variance extracted from the Hessian matrix for sex s and simulation m.

We also present the confidence interval (CI) coverage to evaluate whether variance is correctly estimated by the model. The 95% CI coverage is derived as the ratio of simulations in which the 95% log‐normal CI (which is normal on the link scale) contains the true adult abundance.

### B.2 Results

**TABLE B1:** Model‐based estimates for the standard error of adult abundance estimates, averaged over 1000 simulations (${\bar{\sigma }}_{s}^{N}$), the empirical standard errors of adult abundance estimates, derived from 1000 simulations ($\sigma_{s}^{N}$), the difference between the two ($\Delta \sigma_{s}^{N}$), and the difference relative to the empirical standard error ($\Delta \sigma_{s}^{N}/\sigma_{s}^{N}$; %), for both sexes of the simple species.

Scenario	Males				Females			
	${\bar{\sigma }}_{♂}^{N}$	$\sigma_{♂}^{N}$	$\Delta \sigma_{♂}^{N}$	$\Delta \sigma_{♂}^{N}/\sigma_{♂}^{N}$	${\bar{\sigma }}_{♀}^{N}$	$\sigma_{♀}^{N}$	$\Delta \sigma_{♀}^{N}$	$\Delta \sigma_{♀}^{N}/\sigma_{♀}^{N}$
ME‐67:GC + 5	126.84	130.20	−3.36	−2.58	128.27	136.09	−7.82	−5.75
ME‐67:GC + 10	65.96	86.14	−20.18	−23.43	66.49	87.54	−21.05	−24.05
ME‐33:GC + 0	183.04	182.56	0.49	0.27	185.51	192.59	−7.08	−3.68
ME‐33:GC + 5	126.75	129.92	−3.17	−2.44	128.19	135.83	−7.65	−5.63
ME‐33:GC + 10	65.99	85.56	−19.57	−22.87	66.53	87.02	−20.49	−23.54
ME+0:GC‐5	234.63	237.00	−2.37	−1.00	238.17	251.53	−13.36	−5.31
ME+0:GC + 0	182.76	182.35	0.41	0.22	185.23	192.36	−7.14	−3.71
ME+0:GC + 5	126.51	129.46	−2.96	−2.28	127.97	135.38	−7.41	−5.47
ME+0:GC + 10	66.17	84.72	−18.55	−21.89	66.74	86.31	−19.57	−22.67
ME+33:GC‐10	256.19	266.36	−10.17	−3.82	260.46	282.11	−21.66	−7.68
ME+33:GC‐5	232.56	235.40	−2.84	−1.21	236.10	249.59	−13.49	−5.41
ME+33:GC + 0	182.11	181.85	0.27	0.15	184.61	191.81	−7.20	−3.76
ME+33:GC + 5	125.76	128.52	−2.77	−2.15	127.24	134.44	−7.20	−5.35
ME+33:GC + 10	66.15	83.65	−17.50	−20.92	66.74	85.30	−18.56	−21.76
ME+67:GC‐10	251.11	261.46	−10.35	−3.96	255.29	276.67	−21.38	−7.73
ME+67:GC‐5	230.12	233.59	−3.46	−1.48	233.65	247.46	−13.81	−5.58
ME+67:GC + 0	181.04	180.97	0.07	0.04	183.54	190.85	−7.31	−3.83
ME+67:GC + 5	124.62	127.23	−2.62	−2.06	126.11	133.08	−6.97	−5.24
ME+67:GC + 10	65.94	82.44	−16.50	−20.02	66.55	84.13	−17.58	−20.89

*Note*: The first number in a scenario label refers to the assumed measurement error on length; the second number refers to the used growth function. Scenarios ME‐67:GC‐10, ME‐67:GC‐5, ME‐67:GC + 0, ME‐33:GC‐10, ME‐33:GC‐5, and ME+0:GC‐10 were not included as (most of) the simulations did not successfully fit. Scenario 3‐3 used the correct measurement error standard deviation (2.89) and growth curve specification.

**TABLE B2:** Model‐based estimates for the standard error of adult abundance estimates, averaged over 1000 simulations (${\bar{\sigma }}_{s}^{N}$), the empirical standard errors of adult abundance estimates, derived from 1000 simulations ($\sigma_{s}^{N}$), the difference between the two ($\Delta \sigma_{s}^{N}$), and the difference relative to the empirical standard error ($\Delta \sigma_{s}^{N}/\sigma_{s}^{N}$; %), for both sexes of the complex species.

Scenario	Males				Females			
	${\bar{\sigma }}_{♂}^{N}$	$\sigma_{♂}^{N}$	$\Delta \sigma_{♂}^{N}$	$\Delta \sigma_{♂}^{N}/\sigma_{♂}^{N}$	${\bar{\sigma }}_{♀}^{N}$	$\sigma_{♀}^{N}$	$\Delta \sigma_{♀}^{N}$	$\Delta \sigma_{♀}^{N}/\sigma_{♀}^{N}$
ME‐67:GC + 5	94.60	103.71	−9.11	−8.78	64.03	73.15	−9.12	−12.47
ME‐67:GC + 10	53.66	74.95	−21.29	−28.40	31.49	55.27	−23.78	−43.02
ME‐33:GC + 0	142.41	148.42	−6.01	−4.05	105.71	109.27	−3.56	−3.26
ME‐33:GC + 5	94.49	103.38	−8.90	−8.61	63.93	72.79	−8.85	−12.16
ME‐33:GC + 10	53.72	74.51	−20.80	−27.91	31.58	54.80	−23.22	−42.37
ME+0:GC‐5	205.94	219.86	−13.92	−6.33	163.31	179.16	−15.85	−8.85
ME+0:GC + 0	141.84	147.82	−5.98	−4.05	105.08	108.88	−3.80	−3.49
ME+0:GC + 5	94.18	102.81	−8.63	−8.40	63.71	72.22	−8.51	−11.78
ME+0:GC + 10	53.75	73.85	−20.10	−27.22	31.70	54.06	−22.36	−41.36
ME+33:GC‐10	269.24	300.08	−30.84	−10.28	221.69	262.50	−40.81	−15.55
ME+33:GC‐5	203.93	217.85	−13.92	−6.39	161.13	177.10	−15.97	−9.02
ME+33:GC + 0	140.97	146.93	−5.97	−4.06	104.17	108.29	−4.12	−3.81
ME+33:GC + 5	93.63	101.96	−8.33	−8.17	63.33	71.47	−8.14	−11.39
ME+33:GC + 10	53.68	72.97	−19.29	−26.44	31.80	53.13	−21.33	−40.15
ME+67:GC‐10	264.83	295.13	−30.30	−10.27	216.85	256.41	−39.57	−15.43
ME+67:GC‐5	201.47	215.43	−13.96	−6.48	158.47	174.64	−16.17	−9.26
ME+67:GC + 0	139.79	145.77	−5.98	−4.10	102.99	107.51	−4.53	−4.21
ME+67:GC + 5	92.89	100.93	−8.05	−7.97	62.82	70.62	−7.81	−11.05
ME+67:GC + 10	53.51	71.95	−18.44	−25.62	31.88	52.10	−20.22	−38.81

*Note*: The first number in a scenario label refers to the assumed measurement error on length; the second number refers to the used growth function. Scenarios ME‐67:GC‐10, ME‐67:GC‐5, ME‐67:GC + 0, ME‐33:GC‐10, ME‐33:GC‐5, and ME+0:GC‐10 were not included as (most of) the simulations did not successfully fit. Scenario 3‐3 used the correct measurement error standard deviation (2.89) and growth curve specification.

**TABLE B3:** The 95% confidence interval (CI) coverage for the sex‐specific adult abundance for the successfully converging scenarios, denoted in percentages (%).

Scenario	Simple species		Complex species	
	${\hat{N}}_{♂}$	${\hat{N}}_{♀}$	${\hat{N}}_{♂}$	${\hat{N}}_{♀}$
ME‐67:GC + 5	58.5	60.0	49.0	30.5
ME‐67:GC + 10	1.6	2.7	2.7	0.8
ME‐33:GC + 0	96.3	96.4	95.9	95.8
ME‐33:GC + 5	58.0	59.9	48.6	30.3
ME‐33:GC + 10	1.5	2.6	2.7	0.8
ME+0:GC‐5	73.2	74.4	49.7	28.4
ME+0:GC + 0	96.1	96.4	95.9	95.4
ME+0:GC + 5	57.7	59.7	48.3	29.9
ME+0:GC + 10	1.7	2.5	2.6	0.7
ME+33:GC‐10	56.4	56.8	4.2	0.7
ME+33:GC‐5	74.7	76.4	51.6	31.0
ME+33:GC + 0	96.1	96.2	95.7	94.8
ME+33:GC + 5	56.3	58.4	47.5	29.0
ME+33:GC + 10	1.7	2.6	2.4	0.6
ME+67:GC‐10	60.7	62.1	5.3	1.1
ME+67:GC‐5	77.1	78.2	54.6	35.4
ME+67:GC + 0	96.3	96.2	95.5	94.4
ME+67:GC + 5	54.4	55.9	46.4	27.8
ME+67:GC + 10	1.5	2.6	2.4	0.4

*Note*: The 95% CIs were estimated for every simulation using the model‐based standard error assuming a normal distribution on the link scale, which results in a log‐normal distribution on the real scale.

## APPENDIX C MODELLING POPULATION GROWTH

### C.1

In an alternative version of this model we included a growth parameter r, which allowed for the estimation of exponential growth or decline in the population size. Including growth in the model meant that the distribution of age given measured length $f\left(a|\ell^{*}\right)$ is no longer defined exclusively by survival parameter ϕ but rather by a combination of ϕ and r (Caswell, 2006; Section 4.5.2.1), given that the population reached a new stable age distribution. Hillary et al. (2018) estimated $f\left(a|\ell^{*}\right)$ by grouping the data and fitting a multinomial distribution; however, this was beyond the scope of our research. We could have assumed that the population had settled into a new equilibrium, in which case the age distribution $f\left(a\right)$ would be proportional to the dominant eigenvector from the associated Leslie matrix. However, as our population growth/decline was stochastic rather than systematic, this did not seem appropriate (see the Discussion for more detail). We did run our simulation study with yearly growth parameters r and r assuming that the $f\left(a|\ell^{*}\right)$ as presented in the main text was *approximately* correct, that is, ignoring population growth in the age distribution formulation. We decided not to include this part of the study in the main body of this manuscript, as we did not believe that the results could be used to accurately assess the effect of incorrect ageing on parameter estimation. Nonetheless, we included these results here for completeness as they could contain some valuable insight and form the basis for future research. As we considered abundance for both sexes separately, we estimated the following four parameters: $N_{,t_{0}}$, $N_{,t_{0}}$, r and r, where $t_{0}$ is some reference year. The kinship probabilities remained the same as presented in the Equations (4)–(7). As population size was no longer assumed equal for all years, abundances in different years are linked through a geometric population dynamics model:

(C1)
$$N_{t}=N_{t_{0}}r^{t-t_{0}},$$

where $r\in \left(0,\infty \right)$ denotes the yearly growth rate. We set $t_{0}=2014$ to match Bradley, Conklin, Papastamatiou, McCauley, Pollock, Kendall, et al. (2017) as closely as possible.

Estimated abundance through time.

We fit our 25 scenarios, consisting of all combinations of 5 different measurement errors and 5 different growth curves, to both populations. We modelled the male and female side of the population separately resulting in four figures, each containing 19 population history plots—six plots are blank since the models in these scenarios did not (all) fit correctly. In Figures C1, C2, C3, C4 we notice a similar pattern of over‐ and underestimation related to shifting the growth curves. However, as we also model exponential growth or decline, we also notice effects of shifting the growth curves on the direction and magnitude of this trend. Albeit potentially informative, due to the inconsistency between modelling growth and the assumed age distribution we believe that these results cannot be directly used for inference.
